# Human PMSCs-derived small extracellular vesicles alleviate neuropathic pain through miR-26a-5p/Wnt5a in SNI mice model

**DOI:** 10.1186/s12974-022-02578-9

**Published:** 2022-09-07

**Authors:** Yitian Lu, Jintao Zhang, Fanning Zeng, Peng Wang, Xiangna Guo, Haitao Wang, Zaisheng Qin, Tao Tao

**Affiliations:** 1 Department of Anesthesiology, Central People’s Hospital of Zhanjiang, Zhanjiang, Guangdong People’s Republic of China; 2 Department of Anesthesiology, Nanfang Hospital, Southern Medical University, Guangzhou, Guangdong People’s Republic of China; 3 Department of Anesthesiology, Zhujiang Hospital, Southern Medical University, Guangzhou, Guangdong People’s Republic of China; 4 School of Pharmaceutical Sciences, Southern Medical University, Guangzhou, Guangdong People’s Republic of China

**Keywords:** Neuropathic pain, hPMSCs, sEVs, miR-26a-5p, Wnt5a, Neuroinflammation

## Abstract

**Background:**

Mesenchymal stem cell (MSCs)-derived small Extracellular Vesicles (sEVs) are considered as a new cell-free therapy for pain caused by nerve injury, but whether human placental mesenchymal stem cell-derived sEVs relieve pain in sciatic nerve injury and its possible mechanism are still unclear. In this study, we investigated the roles of hPMSCs-derived sEVs and related mechanisms in neuropathic pain.

**Methods:**

The spared nerve injury (SNI) mouse model was employed. Intrathecal injection of sEVs or miR-26a-5p agomir was performed on the seventh day of modeling, to study its anti-nociceptive effect. sEVs’ miRNA sequencing (miRNA-Seq) and bioinformatics analysis were performed to study the downstream mechanisms of miRNAs. RT-qPCR, protein assay and immunofluorescence were used for further validation.

**Results:**

A single intrathecal injection of sEVs durably reversed mechanical hypersensitivity in the left hind paw of mice with partial sciatic nerve ligation. Immunofluorescence studies found that PKH26-labeled sEVs were visible in neurons and microglia in the dorsal horn of the ipsilateral L4/5 spinal cord and more enriched in the ipsilateral. According to miRNA-seq results, we found that intrathecal injection of miR-26a-5p agomir, the second high counts microRNA in hPMSCs derived sEVs, significantly suppressed neuropathic pain and neuroinflammation in SNI mice. Bioinformatics analysis and dual-luciferase reporter gene analysis identified Wnt5a as a direct downstream target gene of miR-26a-5p. The results showed that overexpression of miR-26a-5p in vivo could significantly reduce the expression level of Wnt5a. In addition, Foxy5, a mimetic peptide of Wnt5a, can significantly reverse the inhibitory effect of miR-26a-5p on neuroinflammation and neuropathic pain, and at the same time, miR-26a-5p can rescue the effect of Foxy5 by overexpression.

**Conclusions:**

We reported that hPMSCs derived sEVs as a promising therapy for nerve injury induced neuropathic pain. In addition, we showed that the miR-26a-5p in the sEVs regulated Wnt5a/Ryk/CaMKII/NFAT partly take part in the analgesia through anti-neuroinflammation, which suggests an alleviating pain effect through non-canonical Wnt signaling pathway in neuropathic pain model in vivo.

**Supplementary Information:**

The online version contains supplementary material available at 10.1186/s12974-022-02578-9.

## Background

Mesenchymal stem cell (MSC) is a type of cell which can self-renew and differentiate into various cell lineages. In the past decades, MSCs derived from various tissues have been found that could produce therapeutic effect through their differentiation and/or secreting function [1–5]. Compared with other tissues, human placental is an ideal MSCs reservoir which could obtain MSCs non-invasively and elicit few ethic concerns [3, 6–10]. Previously, human placenta-derived mesenchymal stem cell (hPMSC) transplantation has been reported could recover ovarian function [11], protect brain function after cerebral ischemic–reperfusion injury [12], attenuate spinal cord injury [13]. However, MSCs transplantation therapy has been limited by cell retention rates and potential risks in tumorigenesis. In addition, more importantly, many studies found that the therapeutic effects are mainly attributed to the MSCs’ secreting function, such as cytokines, growth factors, and small Extracellular Vesicles (sEVs) [1, 4, 14–18].

sEVs can serve as a means of intercellular communication by facilitating the transfer of various RNA, DNA, cytokines, and growth factors between cells [19]. Currently, sEVs of MSCs derived from different sources, e.g., human umbilical cord blood, adipose, bone marrow, have been demonstrated its therapeutic potential in wound healing, myocardial injury, spinal cord injury etc. in preclinical and clinical investigations [20, 21]. Shiue et al. reported that sEVs from umbilical cord MSCs alleviated induced by nerve injury-induced allodynia [4]. Since hPMSCs have been identified in human placenta and confirmed its multilineage differentiation potential in 2004 [22], research focused on diseases therapy based on hPMSCs and its sEVs gradually increased. Apart from hPMSCs could produce directly therapeutic effect in diseases, sEVs derived from hPMSCs also exhibit therapeutic potential. hPMSCs could secret sEVs to regulate cerebral blood flow in the post-MCAO brain and ameliorate neurological function [2], and miR-21 in hPMSCs derived sEVs can also activate exogenous antioxidant defenses via PTEN/PI3K–Nrf2 axis to attenuate the senescence of CD4^+^ T cells [19]. However, to the best of our knowledge, there are no reports investigate the biological and therapeutic effects of hPMSCs derived sEVs in neuropathic pain.

Neuropathic pain is a disorder of somatic dysfunction which is induced by peripheral nerve damage and is characterized by allodynia and hyperalgesia [23]. The underlying pathophysiology of neuropathic pain involves peripheral and central sensitivity. The basis of nociceptive pathway sensitization includes alterations in ion channels, glial-derived mediators, activation of immune cells, and epigenetic regulation [24]. Neuropathic pain is estimated to affect 7–10% of the general population, and its incidence is likely to increase due to an aging global population, an increasing diabetes incidence, and an elevating cancer survival rates [25]. According to reports, chronic pain is estimated to affect more than 100 million adults in the United States and cost more than $600 billion annually [1]. It remains a serious public health problem today [26, 27].

Many procedures have been developed to treat neuropathic pain, including medication (e.g., opioids, NASIDs, and ion channel inhibitors), invasive therapy (e.g., nerve blocks, radiofrequency ablation, and spinal cord simulation) or medication combined with invasive therapy over the past decades [25]. Unfortunately, there are no therapeutic strategies could alleviate neuropathic pain efficiently, especially for refractory chronic pain. MSCs derived sEVs exhibited a promising therapeutic potential in different chronic pain models [1]. In the models of spinal cord injury, hMSCs-derived EVs exerted a protective role through their contents of miR-21 and miR-19b [28]. Besides, sEVs derived from human umbilical cord mesenchymal stem cell (UCMSC) have been used as a cell-free therapy for spinal nerve injury-induced pain in rats. SEVs treatment suppressed nerve ligation-induced neuroinflammation in spinal cord and DRG (dorsal root ganglion). It also inhibited the level of TNF-α and IL-1β in the dorsal root ganglion of nerve-ligated rats. It was speculated that miRNA among the UCMSC played a key role in analgesia [4]. Previous studies also showed hPMSCs derived sEVs anti-inflammation, anti-oxidative effects; however, whether their sEVs could alleviate neuropathic pain has not been reported yet.

Therefore, in the current study, we examined the effect and target cell types of hPMSCs derived sEVs in nerve injury induced neuropathic pain. Furthermore, we investigated the underlying mechanism of hPMSCs derived sEVs’ analgesia effect through RNA-seq. Finally, we demonstrated that miR-26a-5p and its target gene, Wnt5a, play vital role in alleviating allodynia through anti-inflammation in spinal dorsal horn.

## Methods

### Animal and pain models

Male C57/BL6 mice (8 weeks, 22 ± 2 g) were purchased from the central animal facility of Southern Medical University (Guangzhou, China). They were housed under standard conditions of light and dark cycles (12 h: 12 h, temperature 25 ℃), free access to food and water.

The spared nerve injury (SNI) model was performed as previously described [29]. Briefly, after mice were anesthetized with sevoflurane (2–5%), the sciatic nerve near the thigh region were isolated and its branch, the sural, common peroneal and tibial nerves were exposed and separated. The common peroneal and tibial nerves were tightly ligated with 4–0 silk at the trifurcation and then were cut at the distal of silk knot, followed by removing 3–5 mm of the distal nerves end. The sural nerve was carefully leaving intact during the surgery. For mice in sham group, the sciatic nerve was isolated and exposed in the same way only without ligated and cut its branches. All the animal studies were carried out according to the approved protocols and guidelines of the Institutional Animal Ethical Care Committee of Southern Medical University Experimental Animal Centre.

### Mesenchymal stem cell culture and identification

Human placental mesenchymal stem cells (hPMSCs) were purchased from Guangzhou Celera Stem Cell Technology Co. Ltd. (Guangzhou, China). hPMSCs were cultured in MSC growth medium, and the medium was changed to serum-free Dulbecco’s modified Eagle’s medium (D-MEM; Thermo Fisher Scientific, Waltham, MA, USA) when the cells reached to about 80% confluence. The conditioned medium was collected after 48 h of incubation. hPMSCs were analyzed by flow cytometry for cell surface antigens, including cluster of differentiation HLA-DR, CD11b, CD19, CD34, CD45, CD73, CD90 and CD105 to identify the phenotype of hPMSCs. In addition, to evaluate the differentiation potential of hPMSCs, osteogenic, adipogenic differentiation was induced using osteogenic, adipogenic differentiation media, respectively.

### SEVs of mesenchymal stem cell isolation and identification

Conditioned medium was centrifuged at 2000×*g* for 10 min at 4 °C. The supernatant was next passed through a 0.2 μm filter (Steradisc; Kurabo, Bio-Medical Department, Tokyo, Japan). Next, the filtrate was ultracentrifuged at 100,000×*g* for 70 min at 4 °C (Optima XE-90 ultracentrifuge with swing rotor, SW41Ti; Beckman Coulter, Brea, CA, USA). The pellet was next washed with PBS and ultracentrifuged at 100, 000 × g for 70 min at 4 °C. The sEVs-enriched fractions were next reconstituted in PBS or D-MEM for further study.

The particle size and concentration of the exocrine body were analyzed by Malvern nanoparticle tracking analyzer NanoSightNS300, and the precipitate of the isolated exocrine body was heavily suspended by 1 ml PBS and sealed on the ice. Select a clean sample pool and wipe the light with clean paper to ensure that no particles are attached to the outer tube wall on the light path, slowly inject the exocrine solution to avoid bubbles, tilt the sample pool moderately, and seal the sample pool with a cover. Put the sample pool into the instrument and operate the instrument test in accordance with the standard operating procedures.

The prepared exocrine suspension 20 μL was loaded on the surface of the copper mesh, and the 1 min was placed at room temperature. The 5 min was carefully dripped with 20 g/L phosphotungstic acid from the side with filter paper at room temperature, and the copper screen was carefully dried with double distilled water from the side with filter paper. After drying at room temperature, it was observed and photographed by transmission electron microscope (Hitachi H-7100 microscope; Hitachi High-Technologies Corporation, Tokyo, Japan).

### SEVs labelling

To track the uptake of hPMSCs-derived sEVs by spinal cord, sEVs’s membrane was labelled with PKH26 Red Fluorescent Cell Membrane Linker Kit (Sigma-Aldrich; PKH26GL-1KT), prior to intrathecal injection of sEVs to the spinal cord, according to the manufacturer’s protocol. Briefly, following incubation with PKH26, the sEVs were washed three times with PBS by ultracentrifugation at 100,000×*g* for 10 min at 4 °C to remove the unbound stain.

### MicroRNA agomir treatment in vivo

hsa-miR-26a-5p agomir, agomir-NC and miR-26a-5p mimic, mimic-NC were all purchased from GenePharma Co. Ltd. (Shanghai, China). The hsa-miR-26a-5p agomir dissolved concentration was 200 nmol/ml in DEPC-treated Water.

### Intrathecal injections

Sevoflurane was used to anesthetize the mouse and fix the mouse position, first locate the tip of the sixth lumbar spinous process (the highest position of the spine), and then insert the micro syringe into the fifth intervertebral space (L5–L6). After the observation of tail twists, a 10 μL volume of miRNA agomir solution was delivered into the body. Then keep the needle in a specific position for 10 s and pull it out slowly to prevent the injected liquid from flowing out.

### Behavioral test

The classic von Frey monofilament (Semmes Weinstein) up and down method was used [30]. After an adaptation period of 1 h, the mice were placed in a plexiglass box on the grid (9 × 25 × 25 cm). At the beginning of the test, we applied von Frey filament to the lateral plantar surface of the hind paw. Bend the filament and hold for 3 s. During behavioral testing, researchers was blinded to treatment groups. In all measurements, measure the threshold force required to cause the hind paw to retract each time and take the average value (the interval between the two measurements is at least 20 min).

### RNA-Seq of sEVs contained microRNA and bioinformatic analysis

miRNAs contained in hPMSCs-derived sEVs were determined by Illumina HiSeq platform. In brief, total RNA from sEVs was prepared and quantified with a NanoDrop ND-2000 (NanoDrop Technologies). RNA integrity was assessed using a 2100 Bioanalyzer (Agilent Technologies, Santa Clara, CA, USA). A total amount of 3 µg total RNA per sample was used as input material for the small RNA library. Small RNA adapters were then ligated to the 5′ and 3′ ends of total RNA. After cDNA synthesis and amplification, the PCR-amplified fragments were purified from the PAGE gel, and the completed cDNA libraries were quantified by the Agilent 2100 bioanalyzer using DNA 1000 Chips. Sequencing libraries were pooled into a single sequencing lane and sequenced using an Illumina HiSeq4000 analyzer.

ENCORI, an encyclopedia of RNA Interactomes was used in the our study to predict the target of miR-26a-5p [31]. The putative target genes of miR-26a-5p were predicted from the following databases: microT, miRanda, PicTar and TargetScan. Venn diagrams were used to screen the intersection of predicted target genes from the four databases. Putative target genes predicted by all four databases will be included in further gene ontology enrichment analysis. The bioinformatics data was analyzed using the DAVID Bioinformatics Resources 6.82 for Gene ontology enrichment [32]. STRING v11 was used for protein interaction network analysis [33]. Statistical analysis and visualization are carried out in R 3.6.3 version Involved R packages: cluster Profiler package (for enrichment analysis and visualization), ggplot2 package (for visualization).

### Dual-luciferase reporter assay

HEK293T cells were seeded at 50% confluence 24 h prior to transfection. Wild-type (WT) or mutant (MUT) Wnt5a 3’-UTR reporter constructs were co-transfected along with an miR-26a-5p-mimic or negative control (NC) using Lipofectamine 2000 (Invitrogen). Take an appropriate amount of Renilla luciferase detection buffer, add 100 μl firefly luciferase detection reagent when measuring each sample, and measure RLU (relative light unit) after mixing. Reporter cell lysates were used as blank controls. When Renilla luciferase was used as an internal reference, the RLU value obtained by the firefly luciferase assay was divided by the RLU value obtained by the Renilla luciferase assay. According to the obtained ratio, compare the activation degree of target reporter gene between different samples.

### RNA extraction and quantitative real-time polymerase chain reaction (qRT-PCR)

Total RNA isolated from spinal cord tissue using TRIzol reagent (Thermo Fisher Scientific, USA) was used for the subsequent qPCR verification. In brief, total RNA samples were used for cDNA library preparation using a PrimeScript RT reagent Kit (TaKaRa, China). mRNA expression was determined by quantitative real-time polymerase chain reaction (qRT-PCR) using a SYBR Premix Ex TaqTM II (Tli RNaseH Plus) kit (TaKaRa, Dalian, China). qRT-PCR was performed on the ABI QuantStudio 6 flex (Applied Biosystems, USA). The PCR reaction was performed as follows: Cycling conditions began with an initial DNA denaturation step at 95 °C for 20 s, followed by 40 cycles at 94 °C for 15 s, 56 °C for 30 s, and 72 °C for 25 s. Glyceraldehyde 3-phosphate dehydrogenase (GAPDH), was the normalization for quantification of gene expression, using the ΔΔCt method. The primers for mouse genes were synthesized by RiboBio (GuangZhou, China), and sequences can be found in Table 1.

**Table 1:** Sequence of primers

Gene	Primer sequences (5′–3′)	
	Forward	Reverse
GAPDH	TTGCTGTTGAAGTCGCAGGAG	TGTGTCCGTCGTGGATCTGA
Ryk	AGCCTTGGACAAAAACACTAGC	GCAAACCCCTACACTGATGTAA
Wnt5a	CAACTGGCAGGACTTTCTCAA	CATCTCCGATGCCGGAACT
CaMKII	TATCCGCATCACTCAGTACCTG	GAAGTGGACGATCTGCCATTT
NFAT	CAGTGTGACCGAAGATACCTGG	TCGAGACTTGATAGGGACCCC

### Western blotting

Mouse spinal cord L4–5 samples were collected, digested with RIPA extraction buffer (Beyotime, China), and then protein samples were separated by 10% SDS–PAGE and transferred to PVDF (Bio-Rad, USA) in a tank transfer system (Bio-Rad, USA). polyvinylidene fluoride, USA) film. Membranes were blocked with 5% non-fat milk in buffer containing 0.1% Tween-20 (TBST) for 1 h, washed 3 times in TBST, and incubated overnight at 4 ℃ with primary antibodies including rabbit anti-Wnt5a (1:1000 dilution; Proteintech; USA; 55184-1-AP), rabbit anti-Ryk (1:1000 dilution; Proteintech; USA; 22138-1-AP), rabbit anti-CaMKII (1:1000 dilution; Proteintech; USA; 13730-1-AP), rabbit anti-NFAT (1:1000 dilution; Proteintech; US; 22023-1-AP), rabbit anti-GAPDH (1:10,000 dilution; Proteintech; USA; 10494-1-AP). After incubation with HRP-conjugated goat anti-rabbit IgG (1:5000 dilution; Bioss; China; Cat#bs-0295G-HRP, AB_10923693), immunoreactive bands were detected by enhanced chemiluminescence (Millipore, USA). The intensity of each band was determined using Image J software 1.8.0 (National Institutes of Health, Bethesda, MA, USA).

### Immunofluorescence staining

Ipsilateral L4–5 spinal cord samples were taken 1–2 h after surgery (sEVs tracking) and on days 7/8/9 (glial cell analysis). Sections were incubated with diluted primary antibodies against Iba1(Wako 559-24761), GFAP (Proteintech 16825-1-AP), MAP2 (Proteintech 17490-1-AP). Sections were then incubated with appropriate secondary antibodies (1:500, Alexa Fluor 488-labeled goat anti-rabbit, mouse IgG; Jackson Immuno Research, West Grove, PA, USA) for 1 h at room temperature. Finally, the slides were mounted with anti-quenching DAPI (49,6-diamino-2-phenylindole) fluorescent mounting medium. Images were acquired with a confocal laser scanning microscope system (Zeiss, LSM 980, Germany) and upright manual fluorescence microscope (Zeiss, ImagerD2, Germany), then processed with Adobe Photoshop 8.0 software (Adobe Systems, Mountain View, CA, USA). Semi-quantitative analysis of fluorescence images of IBA1, GFAP was performed with ImageJ software 1.8.0 (National Institutes of Health, Bethesda, MA, USA). The original image was divided into channels, and the green channel was converted into an 8-bit black and white image, and then the integral gray value of the target area was divided by the area of the target area to obtain the average gray value, which represented the average protein expression in the target area.

### Statistical analysis

Statistical analyses were performed using SPSS 22.0 Statistics (IBM SPSS Statistics for Version 22.0, IBM Corp, North Castle, NY, USA). All data are presented as mean ± SEM. Two-way ANOVA with repeated measures was used to compare the behavioral data of different groups, the data of Western blots and qRT-PCR. Differences were considered to be statistically significant when *p* value less than 0.05.

## Results

### Identification of hPMSCs and derived sEVs

**Fig. 1:**
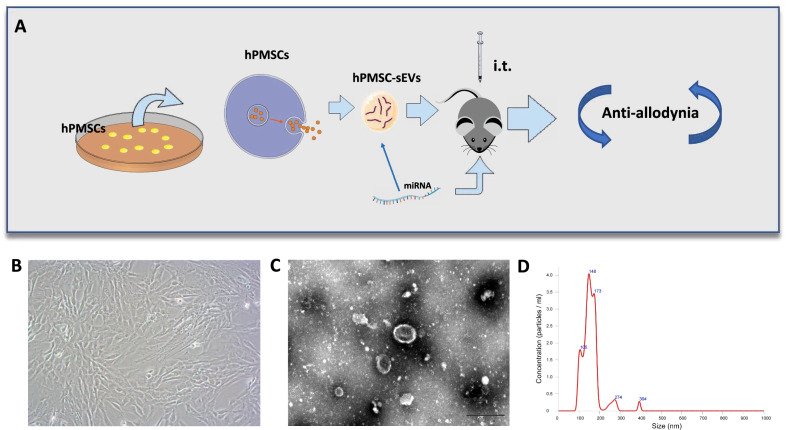
HYPERLINK "sps:id::fig1||locator::gr1||MediaObject::0" Experimental design and characterization of human placental mesenchymal stem cell (hPMSC) sEVs.** A** Intrathecal injection of sEVs derived from human placental mesenchymal stem cells in vitro to induce antinociception in mice. **B** Typical fibroblastic morphology of hPMSCs was verified by microscope (magnification: 100×). **C** Transmission electron microscopy images of sEVs (scale bar: 200 nm). **D** NTA to determine the diameter of hPMSC–sEVs

First, we cultured a batch of human placental mesenchymal stem cells, and the typical fibroblast morphology could be clearly seen under 40 × and 100 × electron microscope (Additional file 1: Fig. S1, Fig. 1B). We analyzed the cell surface antigens of human placental mesenchymal stem cells. The results of flow cytometry showed that the expression rates of CD73, CD90, and CD105 in human placental mesenchymal stem cells were higher than 95%, while the expression rates of CD34, CD19 and CD45, CD11b, and HLA–DR were hardly expressed (Additional file 2: Fig. S2). We centrifuge and filter the conditioned medium, followed by ultracentrifugation for 70 min to obtain sEVs. Electron microscopy exhibited revealed typical circular particles ranging from 30 to 200 nm in diameter, and nanoparticle tracking analysis (NTA) revealed a similar size distribution of hPMSC–sEVs (Fig. 1C, D).

### sEVs of hPMSCs alleviates spared nerve injury induced neuropathic pain by a single intrathecal injection

**Fig. 2:**
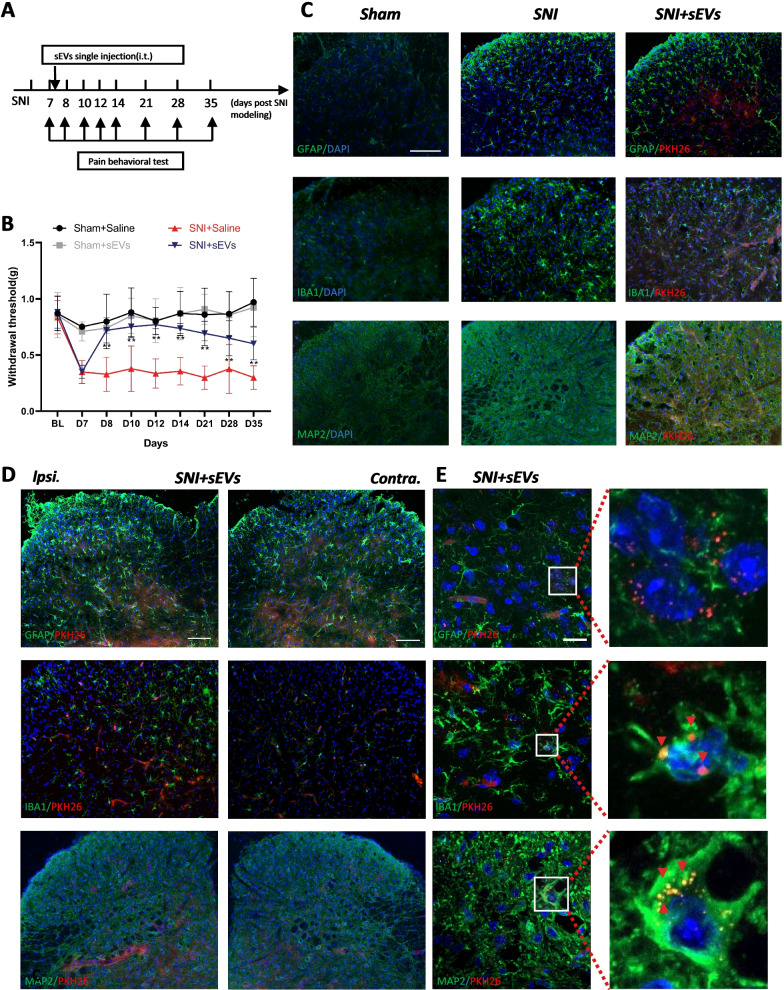
Analgesic effect of hPMSCs sEVs and localization in spinal cord injected intrathecally in mice. **A** Experimental schematic plot for the establishment of the mouse model of spared nerve injury (SNI). **B** 50% paw withdraw threshold (PWT) of left hind paw of different treatment groups mice. At the day of 8, 10, 12, 14, 21, 28 and 35, the paw withdrawal thresholds (WTs) of SNI + sEVs group was significantly higher than those of SNI + saline group after single intrathecal injection at the 7th day (**p < 0.01 compared with SNI + saline group by Two-way ANOVA followed by Tukey post hoc test, *n* = 8 in each group). **C** Immunofluorescence study showed GFAP astrocyte/IBA1 microglia/MAP2 neurons (green) in the dorsal horn of the L4–5 spinal cord of SNI mice and changes after intrathecal injection of sEVs (PKH26). The blue spots are DAPI nuclear staining. Scale bar: 50 μm. **D** Immunofluorescent study revealed pkh26 labeled hPMSC sEVs (red) localized within GFAP astrocyte/IBA1 microglia/MAP2 neuron (green) in the L4–5 spinal dorsal horn of SNI mice. The blue spots are DAPI nuclear staining (Scale bar: 100 μm). **E** pkh26 labelled hPMSCs sEVs appeared majorly the in the ipsilateral (Ipsi.) L4–5 spinal dorsal horn (DH) of SNI mice and co-localized mostly with MAP2 neuron. Some sEVs appeared in the IBA1 microglia. Few sEVs could be detected in the GFAP astrocyte. The blue spots are DAPI nuclear staining (Scale bar: 20 μm)

We adopted a spared nerve injury (SNI) model to simulate nerve injury induced neuropathic pain. Then, we evaluated the mechanic threshold at different timepoint (Fig. 2A) and observed a significant decrease in the mice’s affected hindpaw, which reached the minimum threshold at 6–7 days (Fig. 2B). At the seventh day after SNI, we performed a single intrathecal injection of 10 μl hPMSCs derived sEVs at 0.5 μg/μl. Unlike previous study[34], we did not observe an immediately and an early (2 h interval during 6 h after injection) anti-nociceptive effect of sEVs. Instead, we observed a significant increase of mechanic threshold 24 h after intrathecal injection of sEVs when compared with the SNI and saline group, respectively. More appealing, sEVs from hPMSCs could produce a long-lasting analgesia after a single intrathecal injection, which remained a significant difference compared with SNI group till the 35 days. We also found that the activation of microglia was significantly decreased after sEVs injection, while we did not observe in astrocytes (Fig. 2C, D; Additional file 3: Fig. S3).

### hPMSCs sEVs enriched in the ipsilateral dorsal horn of spinal cord and in SNI-activated neuron and microglia

To determine the localization of injected sEVs, we tracked the PKH26–Red–SEVs and co-stained with MAP2, GFAP and IBA1, respectively, in spinal cord in mice 7 days after SNI. We found that sEVs could be found in both side of the spinal cord, whereas sEVs are more enriched in the ipsilateral (Fig. 2D). In addition, we also found that sEVs are more adjacent to the neurons and microglia (Fig. 2E). We can clearly see the co-localization of sEVs and cells in the 63 × confocal image (Fig. 2E). In neuron (MAP2^+^) and microglia (IBA1^+^), sEVs were co-localized with cells, but sEVs were mostly enriched in neuron and microglia cytoplasm, while sEVs in microglia are relatively less in neuron. By switching different fields of view, we did not find the co-localization of sEVs and astrocytes (Fig. 2E). These staining results can indicate that sEVs are mainly cell-specific, most of them are endocytosed by neurons and microglia.

### Bioinformatic results of microRNAs in hPMSCs sEVs

**Fig. 3:**
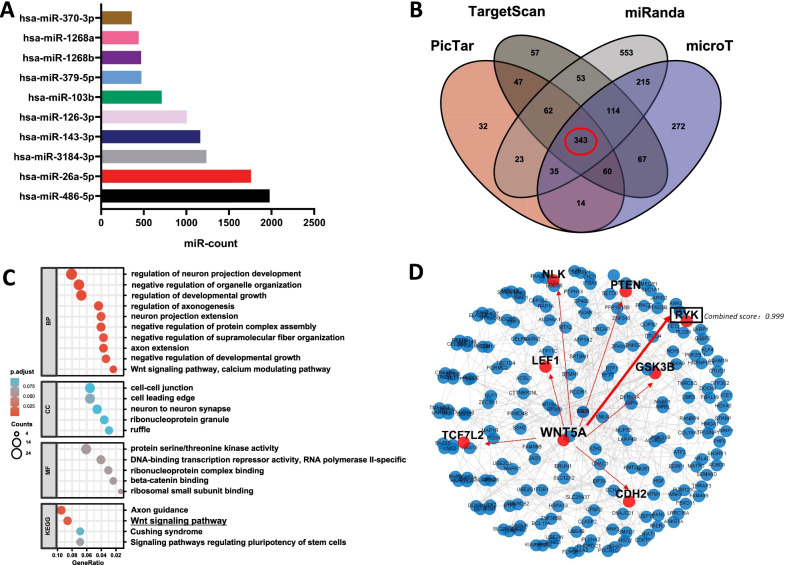
Bioinformatics analysis of miR-26a-5p target genes. **A** miRNA sequencing reads of hPMSCs sEVs. **B** Venn diagram showing target mRNAs for miR-26a-5p from four databases. **C** Gene ontology enrichment analysis of target genes for miR-26a-5p involved in cellular components, biological processes, molecular functions, or KEGG. **D** String functional protein association network of Wnt5a

Many previous data and studies have shown that miRNAs in sEVs play an important role in the process of sEVs acting on target cells. Therefore, we performed miRNA sequencing on sEVs isolated from hPMSCs. Through sequencing, we found a total of 53 highly expressed miRNAs, of which the top ten expressed are shown in Fig. 3A. Then, hsa-miR-26a-5p, the second highest counts microRNA in hPMSCs derived sEVs and previous have been reported possess anti-nociceptive effect, was selected for further analysis. We used four different miRNA target prediction databases (PicTar, TargetScan, miRanda, DIANA–microT) to predict the putative target genes of hsa-miR-26a-5p, and 343 putative target genes, which can be predicted by all the four databases, were selected for gene ontology and KEGG pathway analysis (Fig. 3B). Among enriched pathways and GO annotations (including synapses), we found that the Wnt signaling pathway was the most related pathway in these entries (Fig. 3C). In addition, we found that most of the entries related to neurons and axons in BP and KEGG contain the Wnt5a gene. Therefore, we hypothesized that hsa-miR-26a-5p exerts downstream effects through Wnt5a to regulate pain. In addition, we performed protein interaction analysis on the target genes through the String v11 database (Fig. 3D), and found Ryk, a possible ligand of Wnt5a which has the highest combined score (0.999), for further validation.

### miR-26a-5p produced antinociceptive effect through decrease inflammatory cytokines

**Fig. 4:**
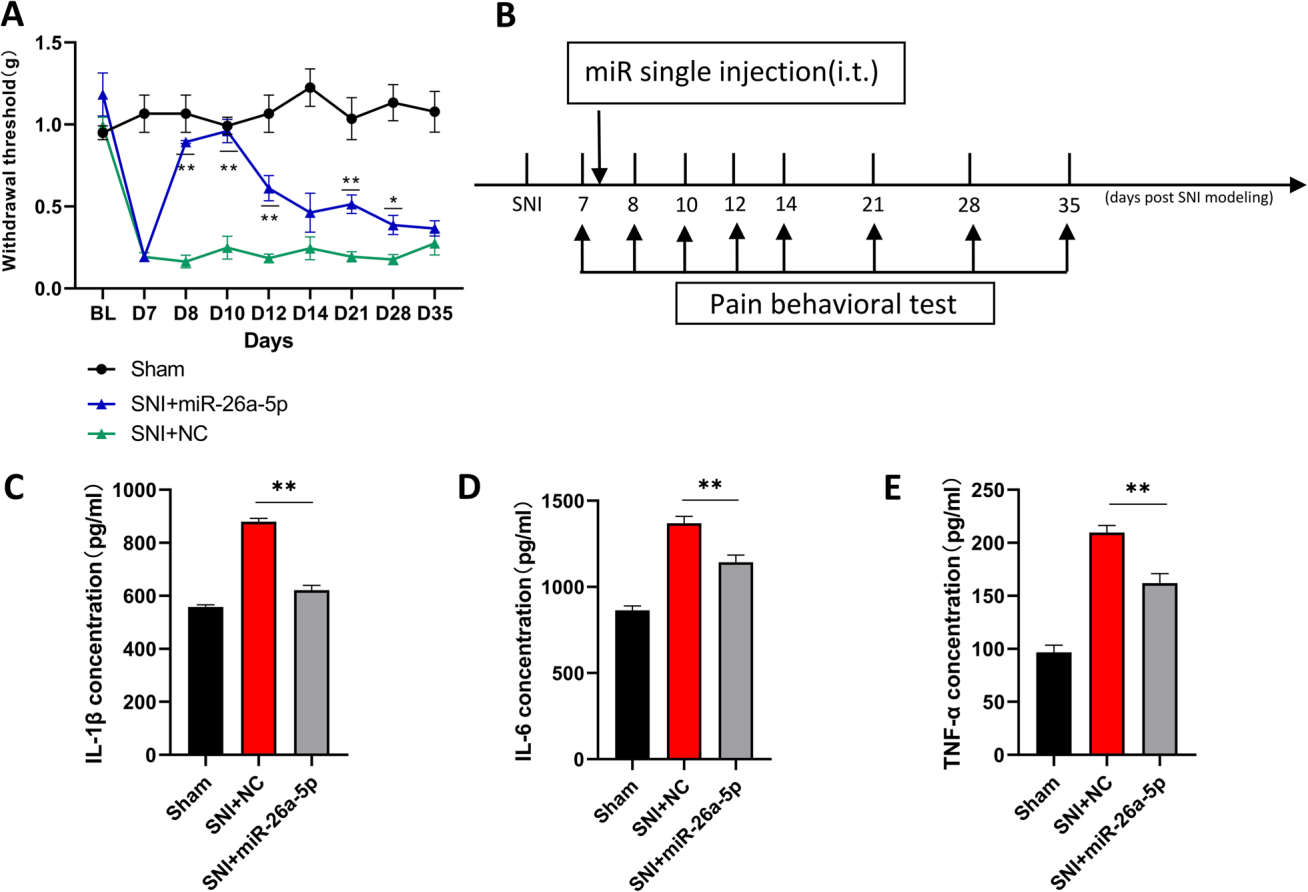
Intrathecal injection of miR-26a-5p reduces SNI-induced pain and relieves inflammation at the spinal cord level. **A** 50% paw withdraw threshold (PWT) of left hind paw of sham, SNI + negative control or miR-26a-5p mice. At the day of 8, 10, 12, 21 and 28, the paw withdrawal thresholds (WTs) of SNI + miR-26a-5p group was significantly higher than those of SNI + NC group after single intrathecal injection at the 7th day (*p < 0.05, **p < 0.01 compared with SNI + NC group by Two-way ANOVA followed by Tukey post hoc test, *n* = 8 in each group). **B** Experimental protocol for establishing the preserved nerve injury (SNI) mouse model and pain behavioral test. **C–E** Protein levels of IL-1β (**C**), IL-6 (**D**), and TNF-α (**E**), in the L4–L5 dorsal spinal cord of mice were tested by ELISA at postoperative day 8. *n* = 6 mice for each group. Data are represented as mean ± sem. **p < 0.01

Next, we performed a single intrathecal injection of hsa-miR-26a-5p agomir (200 nmol/ml, 10 µl per mouse) in SNI mice at the 7th day after surgery (Fig. 4B). Like the hPMSCs derived sEVs, hsa-miR-26a-5p agomir exhibited no immediately and early analgesic effect. However, it produced 3 weeks statistically significant pain-relieving period from the second day after injection (Day 8 to Day 28 after surgery), which reached its peak analgesic effect at Day 10 after surgery, and then decreased gradually till Day 28 after surgery. Although hsa-miR-26a-5p agomir showed a similar pattern in time period and analgesic effect, it cannot reach an equivalent effect as the sEVs, which suggested that other mechanism may take part in the analgesia of the sEVs (Fig. 4A). In addition, one day after intrathecal injection of miR-26a-5p, we found that the level of inflammatory factors including IL-1β (Fig. 4C), IL-6 (Fig. 4D) and TNF-α (Fig. 4E) have a significant decrease compared with the SNI group in the L4–5 spinal cord of mice after surgery. These results suggested that the analgesia of miR-26a-5p may due to its inflammatory suppressive effect through Wnt5 signaling pathway.

### miR-26a-5p directly target Wnt5a in spinal cord of mice

**Fig. 5:**
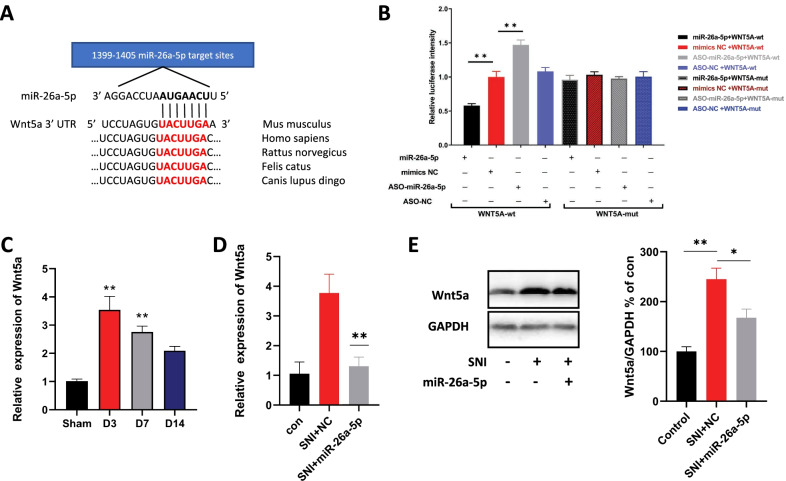
miR-26a-5p targets Wnt5a by binding to the 3’-UTR of Wnt5a mRNA.** A** Bioinformatics analyses of the complementarity of the miR-26a-5p seed sequence to the 3′UTR of Wnt5a and conservation of the putative-binding site in vertebrates. **B** Luciferase reporter assays in HEK293T cells after co-transfection of cells with the wild-type or mutant 3’-UTR of Wnt5a and the miR-26a-5p, NC mimics or ASO-miR-26a-5p, ASO-NC. **C** mRNA levels of Wnt5a on day 3, 7 and 14 after surgery. Statistical significance of mean differences was determined with the Tukey’s multiple comparisons test. **D, E** mRNA(D) and protein(E) levels of Wnt5a in sham, SNI + nc, SNI + miR-26a-5p groups, The SNI + miR group decreased significantly compared with the SNI + NC group, *n* = 6 mice/group, Data are represented as mean ± sem. **p < 0.01

We first confirmed that the miR-26a-5p putative target sequence at the Wnt5a 3′-UTR (3′-untranslated regions) is highly conserved between *Mus musculus* and *Homo sapiens* (Fig. 5A). It is also worth noting that the sequence of miR-26a-5p is identical in *Mus musculus* and *Homo sapiens*. We examined the specific selectivity of miRNA-26a-5p for Wnt5a mRNA by use of a dual-luciferase reporter assay in 293 T cells. The results showed that the Renilla luciferase activity of pmirGLO–Wnt5a–WT transfected cells decreased by about 50% in miR-26a-5p co-transfected cells compared with mimic-NC co-transfected cells; however, expression increased by about 50% in ASO–miR-26a-5p co-transfected cells compared with negative controls. In addition, luciferase levels of pmir–GLO–Wnt5a–Mu transfected cells did not change in miR-26a-5p or ASO–miR-26a-5p co-transfected cells (Fig. 5B). These data suggested that miR-26a-5p directly binds to Wnt5a 3′-UTR. Then we analyzed the expression of Wnt5a mRNA, and our results found that its expression level in the L4–5 segment of spinal cord increased significantly at the 3rd, 7th, and 14th day after surgery compared with sham group (Fig. 5C). We further found that the mRNA and protein expression of Wnt5a was significantly down-regulated one day after intrathecal injection of miR-26a-5p in the L4–5 segment of mice’ spinal cord (Fig. 5D, E). These results demonstrated that hsa-miR-26a-5p could directly target 3'-UTR of Wnt5a and then intervene its expression.

### miR-26a-5p alleviate mechanic allodynia through a non-canonical Wnt signaling pathway

**Fig. 6:**
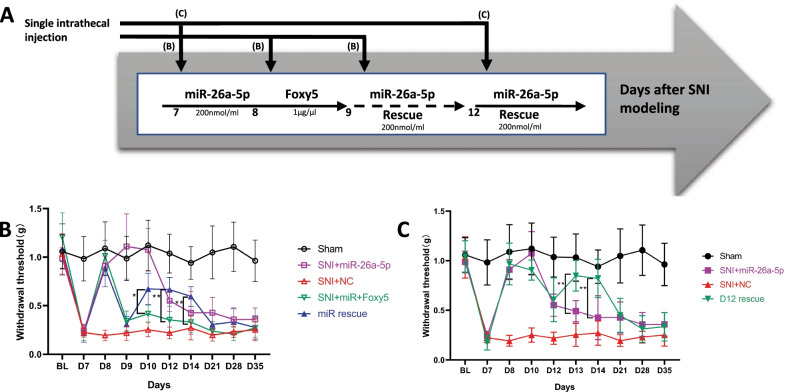
Foxy-5 potentiates nociceptive sensation, while miR-26a-5p rescues this effect. **A** Experimental design and treatment at different timepoints. **B** 50% paw withdraw threshold (PWT) of left hind paw of different treatment groups mice. At the day of 10, 12 and 14, the paw withdrawal thresholds (WTs) of miR-rescue group was significantly higher than those of SNI + miR + Foxy5 group after miR-26a-5p rescue at the 9th day (*p < 0.05, **p < 0.01 compared with SNI + miR + Foxy5 group by Two-way ANOVA followed by Tukey post hoc test, *n* = 8 in each group). **C** At the days of 13 and 14, the paw withdrawal thresholds (WTs) of D12 rescue group was significantly higher than those of SNI + miR-26a-5p group (**p < 0.01 compared with SNI + miR-26a-5p group by Two-way ANOVA followed by Tukey post hoc test, *n* = 8 in each group)

We examined the mechanic threshold at different timepoint after intrathecal injection with hsa-miR-26a-5p agomir, hsa-miR-26a-5p agomir + Foxy5, and a rescue regimen with hsa-miR-26a-5p agomir, Foxy5, and hsa-miR-26a-5p sequentially. Our rescue regimen consisted of intrathecal injection of hsa-miR-26a-5p agomir on the 7th day after SNI, Foxy5(a mimetic peptide of Wnt5a) [35] at the same time on the 8th day after SNI, and hsa-miR-26a-5p agomir again on the 9th day after surgery (Fig. 6A). After intrathecal injection of hsa-miR-26a-5p on 7th day after surgery, the mechanic threshold increased significantly, and when the Foxy5 were injected, the mechanic threshold decreased to the previous level on the next day (Day 9 after SNI). At last, when a hsa-miR-26a-5p were injected again, the mechanic threshold elevated significantly, but threshold did not back to the level after the first hsa-miR-26a-5p injection (Fig. 6B). Furthermore, in the von Frey test in Fig. 4, we found that the analgesic effect of has-miR-26a-5p agomir was only maintained until the 12th day; therefore, we performed another intrathecal injection with the same concentration and dose of has-miR-26a-5p agomir (Fig. 6A, C). The mechanic threshold of the left hindpaw of the mice backing to the level of the sham group as expected on the next day (Day 13 after SNI), but it cannot reach the same level of the first injection (Fig. 6C). Similarly, the maintenance time of this injection is about 2 days or longer (Figs. 4A, 6A, C). These data suggest that Foxy5 can reverse the analgesic effect of has-miR-26a-5p agomir and an additional injection of has-miR-26a-5p agomir also rescues the effect of Foxy5. Furthermore, our results suggest that miR-26a-5p could produce a fast and 3 days period analgesia on SNI mice model, and a second injection could also provide same analgesic period as the first injection, but cannot reach the same analgesic effect.

### miR-26a-5p inhibits spinal cord neuroinflammation in SNI mice through Wnt5a/Ryk/CaMKII/NFAT pathway

**Fig. 7:**
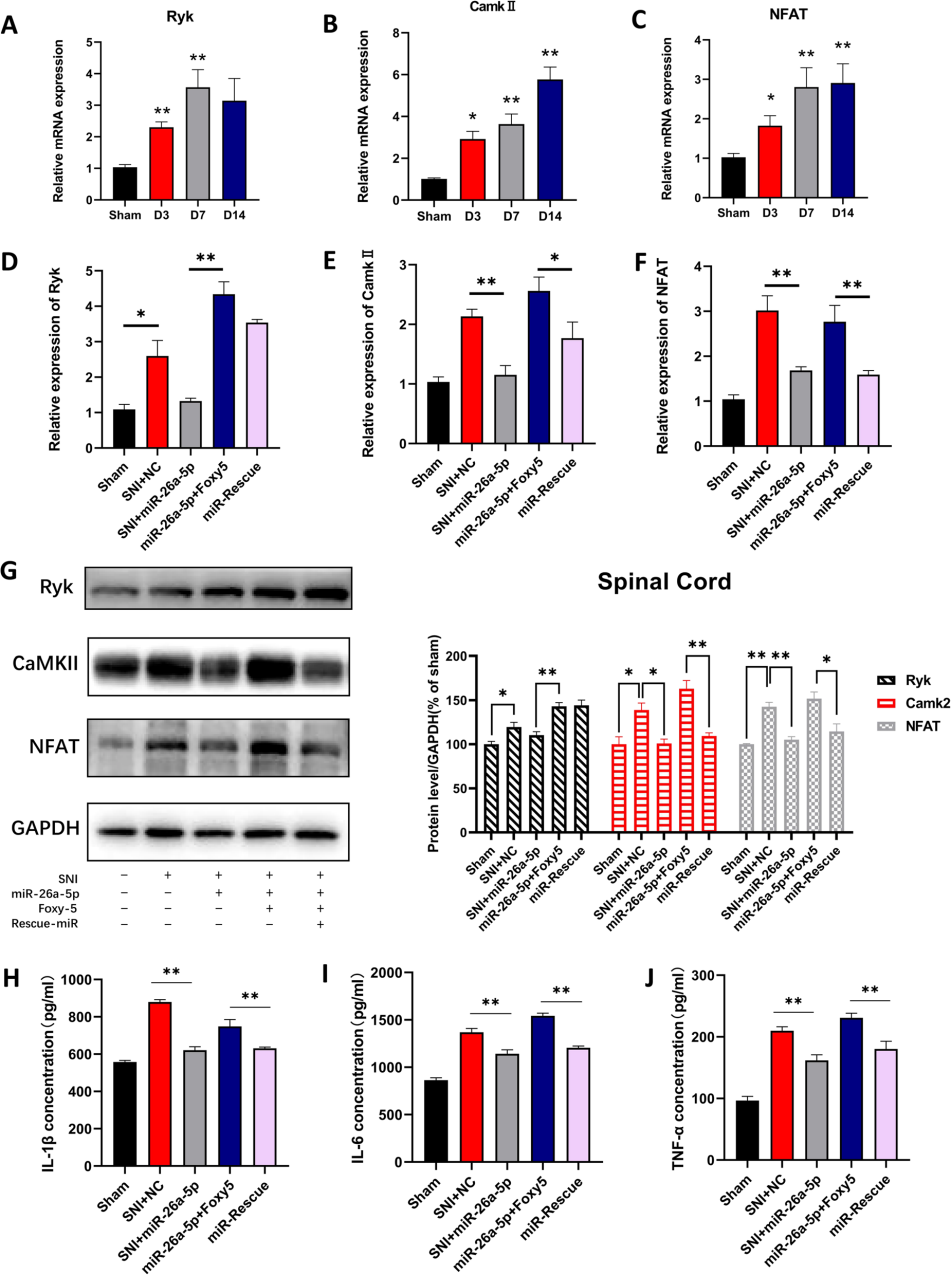
miR-26a-5p alleviate mechanic allodynia through a non-canonical Wnt signaling pathway. **A–C** mRNA levels of Ryk(A), CaMKII (**B**), NFAT(C) on day 3, 7 and 14 after surgery. Data are expressed as fold change compared to sham-operated controls. **D–F** Quantitative RT-PCR was used to assess the mRNA expression level of Ryk(D), CaMKII (E), NFAT(F). **G** Western blot was used to assess the level of Ryk, CaMKII, NFAT after Foxy-5 reverse and miR-26a-5p rescue. Representative immunoblots and quantification showing Foxy-5 reversed the effect of miR-26a-5p, while intrathecal injection of miR-26a-5p one day after Foxy-5 again rescued the effect of Foxy5. Data are expressed as fold change compared to the sham group. **H–J** Protein levels of IL-1β (**H**), IL-6 (**I**), and TNF-α (**J**), in the L4–L5 dorsal spinal cord of mice were tested by ELISA. *n* = 6 mice for each group. Data are represented as mean ± sem. *p < 0.05, **p < 0.01

To explore the downstream of Wnt5a, we investigated the mRNA expression of Ryk, CaMKII and NFAT. Our results found that mRNA of Ryk, CaMKII and NFAT all increased significantly on the 3rd, 7th and 14th day after surgery (Fig. 7A–C), which are consistent with previous studies reported Wnt5a and its downstream genes in non-canonical Wnt pathway [36–39]. Furthermore, we used hsa-miR-26a-5p agomir and Foxy5 (a mimetic peptide of Wnt5a) to elucidate the relationship among Wnt5a, Ryk, CaMKII and NFAT.

Next, we injected hsa-miR-26a-5p agomir intrathecally into mice on the 7th day after modeling. It can be seen that the mRNA and protein expression level of Ryk (Fig. 7D), CaMKII (Fig. 7E, G) and NFAT (Fig. 7F, G) in the L4–L5 spinal cord were significantly reduced on the next day after intrathecal injection of hsa-miR-26a-5p agomir compared with the 7th day of SNI. After intrathecal injection of Foxy5 as an agonist, the levels of CaMKII and NFAT were reversed significantly (Fig. 7E–G). However, Ryk was not significantly changed after injection of hsa-miR-26a-5p agomir at the protein level (Fig. 7G). This may be because the receptors did not change significantly in a short period of time. However, the up-regulation of CaMKII and NFAT by Foxy5 was rescued by another injection of hsa-miR-26a-5p agomir on the 9^th^ day (Fig. 7E–G). We also determined the levels of inflammatory factors in the spinal cord after intrathecal injection of Foxy5. Consistent with our hypothesis, inflammatory factors including IL-1β, IL-6, TNF-α, was significantly upregulated after Foxy5 injection (Fig. 7H–J). hsa-miR-26a-5p agomir can down-regulate the levels of pro-inflammatory factors in the spinal cord and rescue the inflammatory effect of Foxy5. These results suggest that miR-26a-5p may exert anti-inflammatory effects at the spinal cord level through the Wnt5a/Ryk/CaMKII/NFAT signaling pathway.

**Fig. 8:**
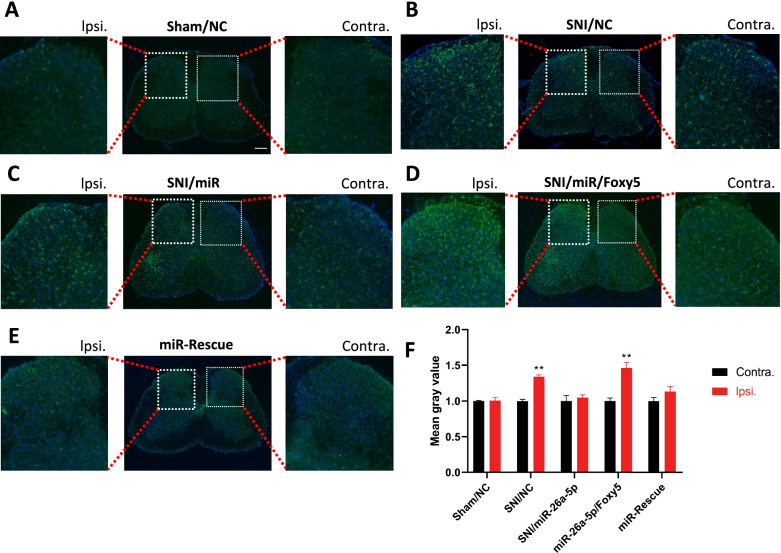
miR-26a-5p inhibits spinal microglia activation in SNI mice. **A–E** Immunofluorescent study revealed IBA1 microglia (green) in the L4–5 spinal dorsal horn of SNI mice. The blue spots are DAPI nuclear staining (Scale bar: 100 μm). **F** Quantification showing the percentage of IBA1 positive microglia was significantly increased in the SNI and miR-26a-5p/Foxy5 group, compared with sham, SNI/miR-26a-5p, miR-rescue group. Data are represented as mean ± sem. **p < 0.01

As can be seen from Figs. 2C and 8B, microglia were significantly activated in the dorsal horn of the ipsilateral spinal cord in SNI mice compared to the contralateral side. After a single intrathecal injection of hsa-miR-26a-5p agomir, immunofluorescence staining of the spinal cord in the L4–L5 segment of the mice showed that the activation of microglia was alleviated compared with the SNI group (Fig. 8C). On the second day after Foxy5 injection, the effect of hsa-miR-26a-5p agomir was completely reversed, and even the activation of microglia exceeded that of SNI/NC mice (Fig. 8D, F). The effect of Foxy5 was rescued by another intrathecal injection of hsa-miR-26a-5p agomir (Fig. 8E). These results all demonstrate from a morphological point of view that miR-26a-5p can inhibit spinal cord neuroinflammation in SNI mice through Wnt5a non-canonical pathway.

## Discussion

In present study, we found that sEVs derived from human placental mesenchymal stem cells (hPMSCs) could alleviated SNI-induced neuropathic pain which may lasting at least 28 days. In addition we further demonstrated that miR-26a-5p in the hPMSCs derived sEVs play an important role in the sEVs analgesic effect through a non-canonical Wnt signaling pathway. We first confirmed that single intrathecal injection hPMSCs derived sEVs could reverse mechanical allodynia in SNI mice and the immunostaining indicated that sEVs decreased the Iba1 expression in dorsal horn of ipsilateral spinal cord, suggesting the analgesic effect of sEVs may relate with inflammatory regulation. Meanwhile, our RNA-seq results and bioinformatic analysis screened a highly conserved microRNA, miR-26a-5p, were enriched in hPMSCs derived sEVs. Further experiments demonstrated that miR-26a-5p could produce similar analgesic effect as hPMSCs derived sEVs. Then, we found that miR-26a-5p could target Wnt5a to regulate Ryk, CaMKII and NFAT expression, which ultimately decreased inflammatory cytokines (IL-1β, IL-6 and TNF-α) in spinal cord. In addition, the analgesic effect of miR-26a-5p could be reversed by Foxy5, a mimicking peptide of Wnt5a. Therefore, our study not only found that hPMSCs derived sEVs could relief nerve injury induced neuropathic pain, but we also demonstrated that these effects are at least partly contributed by the anti-inflammatory effect of miR-26a-5p in sEVs via regulating Wnt5a/Ryk/CaMKII/NFAT signaling pathway.

In recent years, stem cell transplantation has been widely proven to be an effective alternative in chronic pain therapy. Mesenchymal stem cells (MSCs) are a kind of pluripotent stem cells that have multidirectional differentiation and regulate immune function [1], in several chronic animal pain models, they have also been shown to play an analgesic effect. In the CCI model, mesenchymal stem cells derived from adipose tissue have been shown to be effective in reducing chronic neuropathic pain symptoms [40]. Bone marrow mesenchymal stem cells (BMSCs) can suppress neuropathic pain by secreting a variety of biologically active molecules and have highly effective immunomodulatory and immunosuppressive properties [41]. In addition, in osteoarthritis, MSCs have been widely explored as a new approach in the cell therapy of OA, because they have the ability to differentiate into chondrocytes and their immunomodulatory properties [5]. hPMSCs is a new type of MSC which has been identified in 2004. Compared with other MSCs, hPMSCs represent a safe, accessible, abundant, and potentially effective form of stem cell therapy [42, 43]. Placental can be obtained non-invasively when compared with other sources, such as bone marrow [44]. It is also viewed as relatively inexpensive and free of ethical concerns [9]. Previous studies have also compared the superiority of hPMSCs with other MSCs, thus in current study we chose hPMSCs to produce sEVs [6, 45, 46].

MSCs derived sEVs are potential in biotechnology and biomedical research to develop as disease biomarkers as well as therapeutic agents [47]. The up-to-date studies also demonstrated that MSCs’ sEVs is a promising therapeutic strategy for chronic pain. Compared with conventional medicines or procedures, sEVs are derived from the body, so theoretically have better biocompatibility and lower immunogenicity [48]. SEVs communicate biological information with target tissues mainly through receptor-mediated endocytosis, which promotes the internalization of encapsulated contents [48]. In addition, sEVs have strong homing ability and can penetrate biological barriers (such as the blood–brain barrier), so they are ideal targeted drug carriers [49, 50]. However, sEVs directly cultivated from the body may have problems, such as easy rapid clearance in the body and weak targeting, resulting in poor therapeutic effect. The underlying mechanism in current investigations are mainly related with its endosomal content and secretion [1]. SEVs in MSC-conditioned medium (CM) have facilitated the acceleration of fracture healing and it may be mediated by sEVs components, such as microRNAs [51]. Moreover, miR-21 and miR-19b delivered by MSC-derived EVs have been proved to regulate the apoptosis and differentiation of neurons in patients with spinal cord injury [28]. hPMSCs derived sEVs have been proved to play important roles in placenta genesis and pregnancy disorders, Duchenne muscular dystrophy, spinal cord injury, and inflammatory environments [9]. hPMSCs derived sEVs also attenuates CD4^+^ T cell senescence by activating exogenous antioxidant defenses mediated by the PTEN/PI3K–Nrf2 axis via carried miRNA-21 [19]. In addition, the therapeutic effect of hPMSCs derived sEVs are partly attributed to the presence of miR-29c in sEVs [52]. Besides, the increased miR-126 in sEVs released by hPMSCs stimulated by nitric oxide (NO) is believed to contribute to the increased angiogenesis-promoting ability of sEVs [53]. In our study, we found that hPMSCs-derived sEVs could produce a long-lasting and pretty decent pain-relieving period after nerve injury induced neuropathic pain, which may through its anti-inflammatory effect on spinal cord. The immunofluorescent staining showed a reduction in microglia activation, whereas an increased astrocyte reactivity. Consistent with previously reports, MSCs derived sEVs or exosomes could suppress microglia activation and the immunofluorescence quantitative statistical analysis confirmed that the reduced microglia activation is statistical significance (Additional file 3: Fig. S3). Therefore, combined with the decreased inflammatory cytokines after sEVs injection, we speculated that microglia may make major contribution in sEVs analgesic effect. For astrocytes, although the increased reactivity has no statistical significance, a previous study indicated that MSCs derived exosomes normally suppress the astrocyte reactivity [54]. Normally, astrocyte reactivity is increased after different lesions or stress [55]. We think the increased astrocyte reactivity may due to the invasive injection of sEVs in our experiment. However, this interesting phenomenon still need further study to explore potential reasons and mechanism.

As discussed above, miRNAs in the sEVs are one of the main sources of its therapeutic effects [48]. Previous studies showed that pain relieving effect of miRNAs are mainly related with anti-inflammatory, anti-oxidative and autophagy pathway through targeting different genes [56–58]. For example, miR-23a overexpression inhibited the increase of TXNIP and NLRP3 inflammasomes in pSNL mice to relieve neuropathic pain [59]. Studies have shown that the key role of miR-155 in regulating oxaliplatin (OXL)-induced neuropathic pain may through the oxidative stress–TRPA1 signaling pathway [58]. In addition, inhibition of miR-135a-5p reduces autophagy levels and reduces neuropathic pain in CCI rats [56]. In present study, 6 of the top 10 high counts miRNAs have been reported in different pain models or pain diseases, including hsa-miR-486-5p [60], hsa-miR-26a-5p [61], hsa-miR-143-3p [62], hsa-miR-126-3p [63], hsa-miR-103b [64], and hsa-miR-379-5p [65]. Therefore, based on the above research, we hypothesis that miRNAs of hPMSCs derived sEVs may play a vital role in the sEVs neuropathic pain-relieving effect. hsa-miR-486-5p was the highest expressed miRNA in sEVs miRNA sequencing. However, the gene ontology and KEGG pathway enrichment analysis of miR-486-5p predicted target genes does not exhibit a closely relationship with chronic pain (Additional file 4: Fig. S4). Moreover, the hsa-miR-486-5p [60] was only found in the serum of postherpetic neuralgia patients and we did not found other studies reported its relationship with pain. In contrast, miR-26-5p showed a more closely relationship with chronic pain according to bioinformatic analysis and reference. Therefore, we chose hsa-miR-26a-5p, the second high counts miRNA, for further study. Similar to previous report that miR-26a-5p reduced pain in CCI rat models [61], our results confirmed that hsa-miR-26a-5p also could significantly decrease pain in SNI mice model. However, the hsa-miR-26a-5p cannot reach the same pain-relieving level and period as sEVs, which may due to the synergistic and/or additive effect produced by other pain-relieving miRNAs or other contents in the sEVs. Whether an equally analgesic effect and lasing period is produced, though synergistic and/or additive effect among miRNAs in hPMSCs derived sEVs are still need further research.

**Fig. 9:**
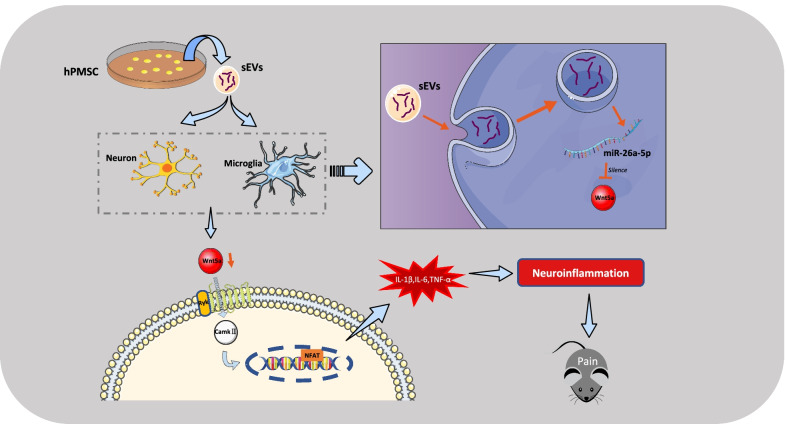
Human PMSCs derived sEVs alleviate neuropathic pain through its anti-inflammatory effect. miR-26a-5p regulated Wnt5a/Ryk/CaMKII/NFAT, a non-canonical Wnt signaling, partly take part in the analgesia of hPMSCs derived sEVs through down-regulating the IL-1β, IL-6, and TNF-α

Wnt signaling is a promising target for neuropathic pain treatment. Previous studies suggested that canonical or non-canonical Wnt pathway, which categorized by whether the downstream depending on the involvement of β-catenin, both play crucial effect through regulating synaptic transmission and plasticity, neuroinflammation, and intraepidermal nerve fiber density in various pain models [39]. In our study, we screened the target genes of miR-26a-5p by bioinformatics analysis, and finally selected the highly enriched non-canonical Wnt pathway ligand Wnt5a for research. We also further confirmed that Wnt5a can be directly target by hsa-miR-26a-5p, and then found that Wnt5a was significantly elevated in the spinal cord in SNI mice model, and pain was also relieved in mice after silencing Wnt5a with miR-26a-5p. Ryk (receptor-like tyrosine kinase) is a canonical Wnt receptor with a Wnt inhibitory factor 1 (WIF1)-like extracellular domain and is capable of interacting with different Wnt ligands [66]. Ryk acts as an important regulator of axonal growth, and studies have demonstrated the potential of Ryk receptors to mediate axonal regeneration in models of neuropathic pain. In addition, studies have shown that Ryk is also a potential therapeutic target for neuropathic pain, which may increase pain sensitivity through mediating excitatory synaptic transmission and CCL2 release (CCR-like protein) [67]. Calcium/calmodulin-dependent protein kinase II (CaMKII) is a multifunctional serine/threonine kinase and is critical for nociceptive transmission and processing [68, 69]. Studies have shown that Ryk activation could subsequently enhance Ca^2+^ dependent CaMKII, which would regulate transcript factor NFAT (Nuclear factor of activated T-cells) [39]. In our study, as showed in Fig. 9, miR-26a-5p downregulated the expression of intracellular CaMKII through the Ryk receptor by silencing Wnt5a, finally affecting the transcription factor NFAT, and then decreased the IL-1β, IL-6, TNF-α level, which provides an alleviating neuropathic pain mechanism through miR-26a-5p/Wnt mediated neuroinflammation (Fig. 9).

## Conclusions

Taken together, we reported that hPMSCs derived sEVs as a promising therapy for nerve injury induced neuropathic pain. Furthermore, we found that microRNAs in hPMSCs derived sEVs’ may mainly contribute to its alleviating allodynia effect. In addition, the miR-26a-5p regulated Wnt5a/Ryk/CaMKII/NFAT, a non-canonical Wnt signaling, at least partly take part in the analgesia of hPMSCs derived sEVs through anti-neuroinflammation, which also provide supplement evidence for chronic pain therapy by targeting non-canonical Wnt signaling pathway.

## Supplementary Information

**Additional file 1.** Supplementary figure S1.

**Additional file 2: Fig. S2. **Surface markers of hPMSCs were tested by FCM. The expression rates of CD73, CD90, and CD105 in human placental mesenchymal stem cells were higher than 95%, while the expression rates of CD34, CD19 and CD45, CD11b, and HLA-DR were hardly expressed

**Additional file 3: Fig. S3**. Quantitative analysis of astrocyte and microglia activation one day after intrathecal injection of sEVs. Quantification showing the percentage of IBA1 positive microglia was significantly increased in the SNI group, compared with sham, SNI+sEVs group. No similar phenomenon was observed in astrocyte SNI+sEVs group. Data are represented as mean ± sem. **p < 0.01.

**Additional file 4: Fig. S4**. Bioinformatics analysis of hsa-miR-486-5p target genes. Gene ontology enrichment analysis of target genes for miR-486-5p involved in biological processes and KEGG.

## Data Availability

The data and materials supporting the conclusions of this study are available from the corresponding author on reasonable request.

## Abbreviations

ANOVA: Analysis of variance
BP: Biological processes
CaMKII: Calmodulin-dependent protein kinase 2
CC: Cellular component
Contra: Contralateral
DAPI: 4′,6-Diamidino-2-phenylindole
DEPC: Diethypyrocarbonate
ELISA: Enzyme-linked immunosorbent assay
FITC: Fluorescein isothiocyanate
GO: Gene ontology
GFAP: Glial fibrillary acidic protein
hPMSCs: Human placental mesenchymal stem cell
Iba1: Ionized calcium-binding adapter molecule 1
i.t.: Intrathecal injection
IL-1β: Interleukin-1β
IL-6: Interleukin-6
KEGG: Kyoto Encyclopedia of Genes and Genomes
Ipsi: Ipsilateral
MF: Molecular function
miRNA-Seq: MiRNA sequencing
MAP2: Microtubule associated protein 2
miRNA: MicroRNA
NFAT: Nuclear factor of activated T cells 1
PPI: Protein–protein interaction
qRT-PCR: Quantitative real-time polymerase chain reaction
Ryk: Receptor-like tyrosine kinase
STRING: Search tool for the retrieval of interacting genes
SNI: Spared nerve injury
SEM: Standard error of the mean
sEVs: Small extracellular vesicles
TBST: Tris-buffered saline and Tween
TNF-α: Tumor necrosis factor-α
Wnt5a: Wingless-type MMTV integration site family, member 5A
