# Supplementary figures and images for: Human PMSCs-derived small extracellular vesicles alleviate neuropathic pain through miR-26a-5p/Wnt5a in SNI mice model
Source: J Neuroinflammation. 2022 Sep 7;19:221. doi: 10.1186/s12974-022-02578-9 (PMC9450435; doi:10.1186/s12974-022-02578-9)

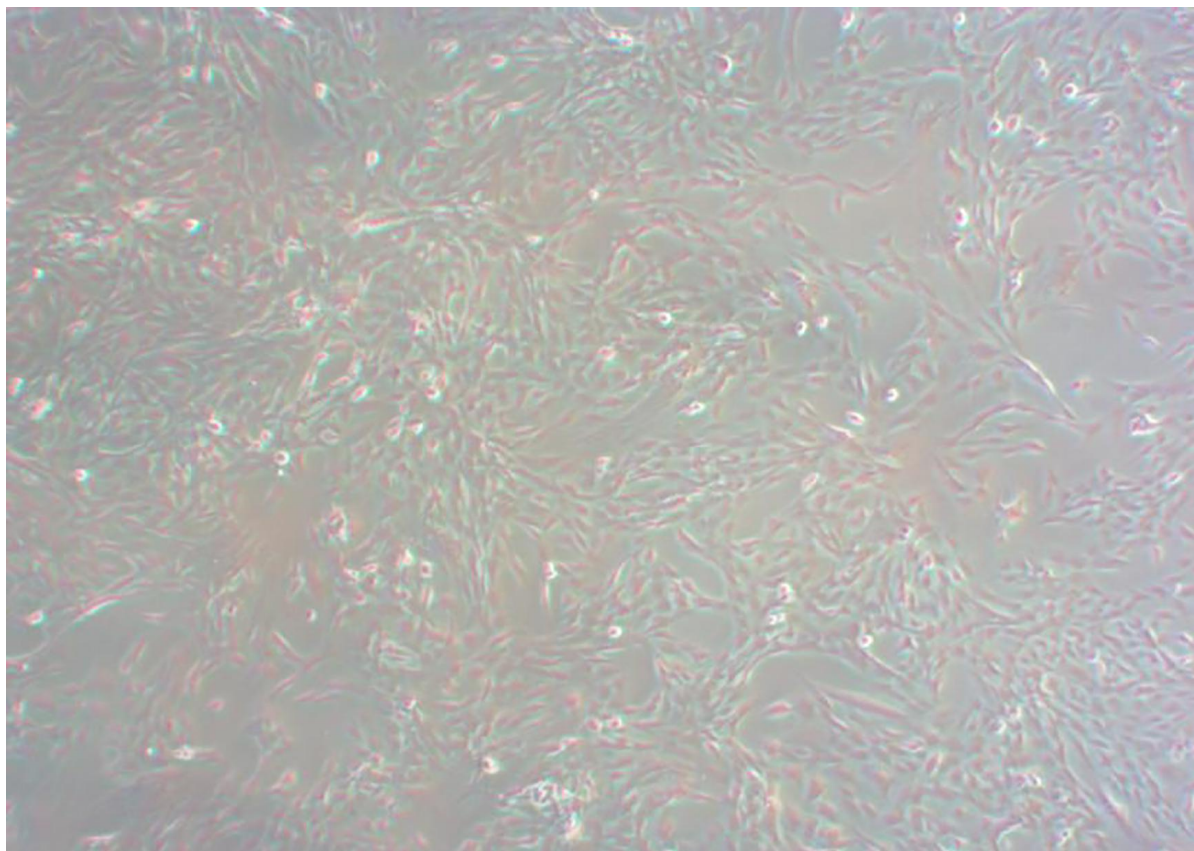

Supplement: Supplementary file 1 — Additional file 1. Supplementary figure S1. [file 12974_2022_2578_MOESM1_ESM.pdf]

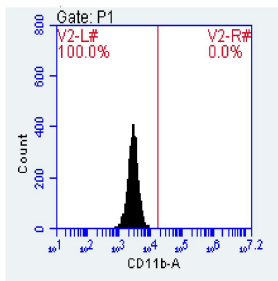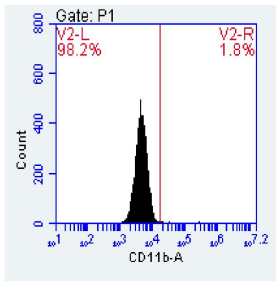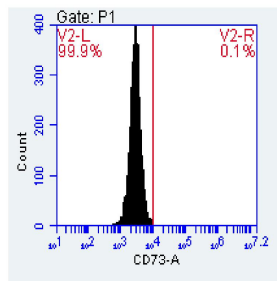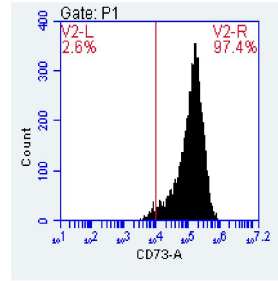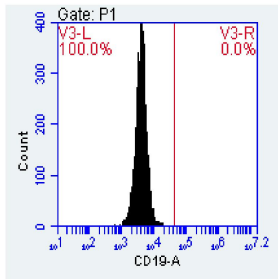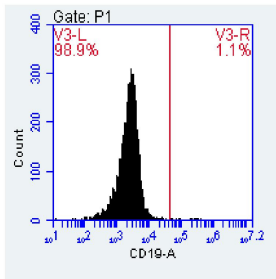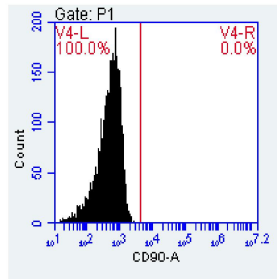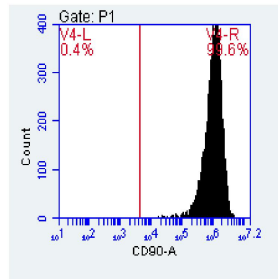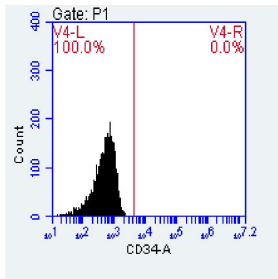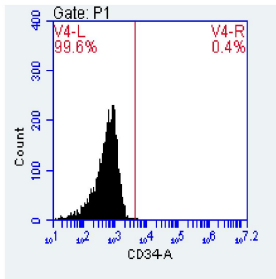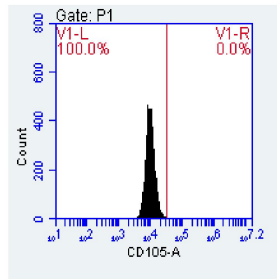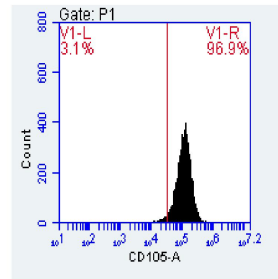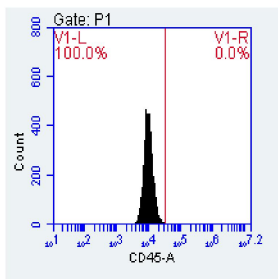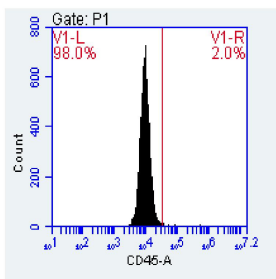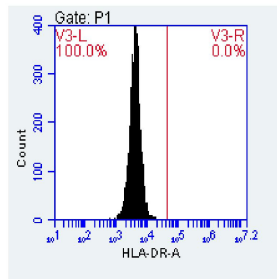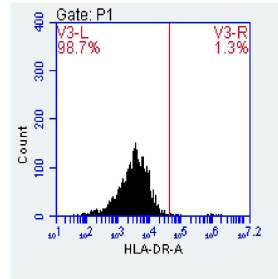

Supplement: Supplementary file 2 — Additional file 2: Fig. S2. Surface markers of hPMSCs were tested by FCM. The expression rates of CD73, CD90, and CD105 in human placental mesenchymal stem cells were higher than 95%, while the expression rates of CD34, CD19 and CD45, CD11b, and HLA-DR were hardly expressed [file 12974_2022_2578_MOESM2_ESM.pdf]

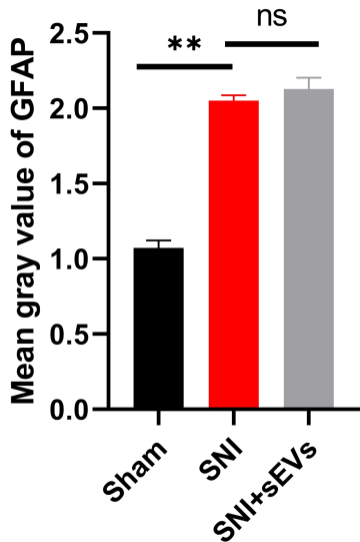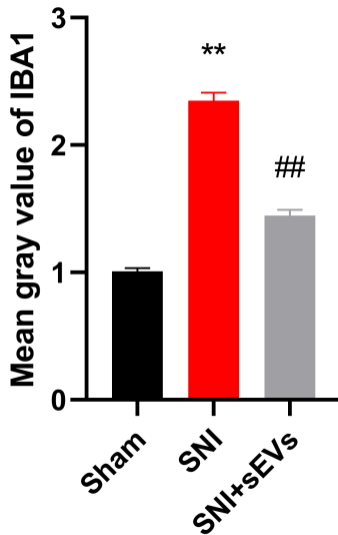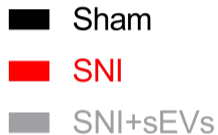

Supplement: Supplementary file 3 — Additional file 3: Fig. S3. Quantitative analysis of astrocyte and microglia activation one day after intrathecal injection of sEVs. Quantification showing the percentage of IBA1 positive microglia was significantly increased in the SNI group, compared with sham, SNI+sEVs group. No similar phenomenon was observed in astrocyte SNI+sEVs group. Data are represented as mean ± sem. **p < 0.01. [file 12974_2022_2578_MOESM3_ESM.pdf]

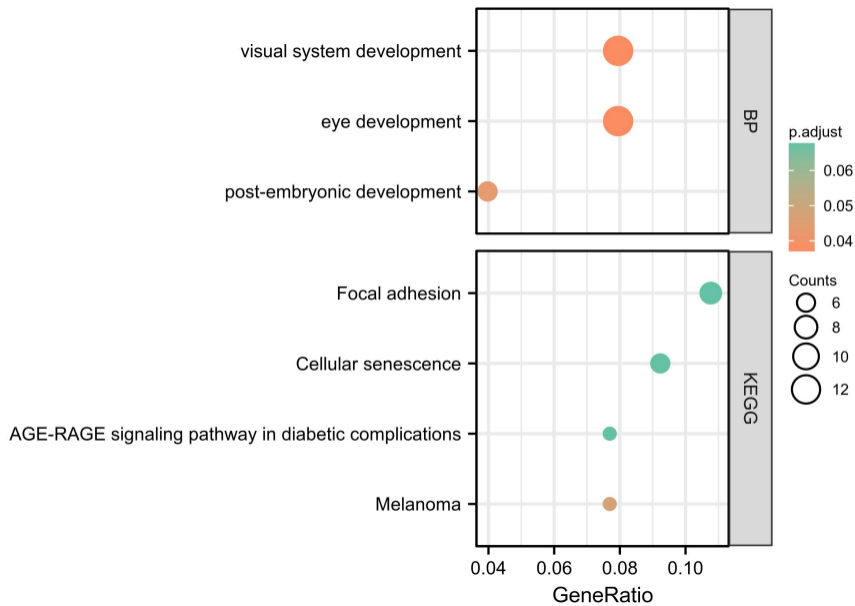

Supplement: Supplementary file 4 — Additional file 4: Fig. S4. Bioinformatics analysis of hsa-miR-486-5p target genes. Gene ontology enrichment analysis of target genes for miR-486-5p involved in biological processes and KEGG. [file 12974_2022_2578_MOESM4_ESM.pdf]
